# Ronald S. Duman, PhD (1954–2020)

**DOI:** 10.1038/s41593-020-0629-3

**Published:** 2020-04-20

**Authors:** Jane R. Taylor, Ralph J. DiLeone, Marina R. Picciotto

**Affiliations:** Department of Psychiatry, Yale University School of Medicine, New Haven, Connecticut USA

## Abstract

Ronald S. Duman, the Elizabeth Mears and House Jameson Professor of Psychiatry, Professor of Neuroscience at the Yale University School of Medicine and Director of the Abraham Ribicoff Research Facilities of the Connecticut Mental Health Center, died unexpectedly of a heart attack on 1 February 2020 while hiking near his home in Guilford, CT. Dr Duman was about to turn 65 years old. He was a member of the US National Academy of Medicine and received many honors for his research on mood disorders, including the Colvin Prize, from the Brain and Behavior Research Foundation, and the Anna-Monika Foundation Prize.

Dr Ronald S. Duman was a varsity middle linebacker at The College of William and Mary, where he received his BS in 1976. He completed his PhD in neuropharmacology with Sam J. Enna at the University of Texas in Houston and then moved to a postdoctoral fellowship with John Tallman. In 1988, Ron joined the faculty in the Department of Psychiatry at Yale University. He and Eric Nestler were assistant professors together when they inaugurated the Laboratory of Molecular Psychiatry with the encouragement of George Heninger and George Aghajanian. This laboratory was a pioneering research program and among the first to focus on the molecular and cellular biology of psychiatric disorders. Ron and Eric were prescient in their realization that studies of molecular mechanisms of psychiatric disorders would have major therapeutic clinical utility.

**Figure:**
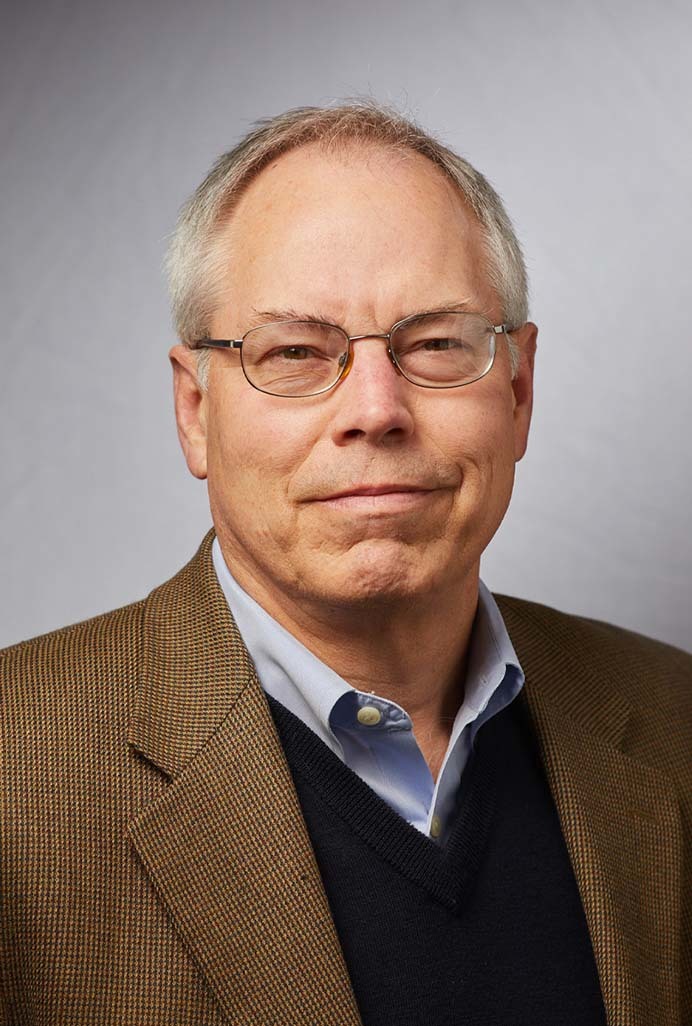
Yale University

The Duman laboratory identified fundamental neurobiological mechanisms underlying the pathological effects of stress on the brain. He was a pioneer in identifying how antidepressant treatments reverse structural and biological changes produced by stress in both animal models and studies of human post-mortem brain tissue. Ron Duman proposed the neurotrophic hypothesis of antidepressant response, identifying deficits in growth factor signaling after chronic or severe stress and the restoration of this signaling by antidepressant treatments. He demonstrated that antidepressants induce adult neurogenesis in the adult hippocampus, and he was the first to identify mTOR signaling and rapid dendritic spine dynamics in prefrontal cortex as key signaling mechanisms responsible for the rapid antidepressant effects of ketamine. Ron’s scientific legacy will live on through the many dozens of graduate students and postdoctoral fellows he mentored and who viewed him as their scientific father. Many of these people came from all over to country to be with Ron’s biological and scientific family at his remembrance and funeral.

Ron Duman was a man of character and integrity. He was also a lifelong football fan (and particularly a Pittsburgh Steelers supporter), and he bonded with many of the trainees who came through the division through a shared love of the game. His legacy as a mentor and role model is reflected in the current and past junior and senior faculty that are part of the Division of Molecular Psychiatry at Yale. His example of carrying out studies with the highest scientific rigor, while maintaining solidarity and support for others, is remarkable and an example of his selfless and generous approach to science.

Every person who knew Ron viewed him as a uniquely kind, honest, generous, humble and thoughtful man. It is fitting, given the focus of his research work on stressors, that he taught those around him how to be resilient through the challenges that inevitably occur over a scientific career. Ron supported many trainees and faculty at the start of their careers by leading from the side. Rather than imposing his views on others, he provided an environment in which others could step up to leadership in their own areas of research. His former trainees now run successful research labs around the world. Ron exemplified persistence and patience in his research as well as his leadership. He was steady, focused, organized, fair and always composed. It will be impossible to fill the void that Ron leaves both scientifically and personally, though we all do our best to both appreciate and emulate these attributes. When faced with a difficult situation, we only need to ask, “what would Ron do?” to set us on the right path.

Ron is survived by his wife, neuroscientist Cathy Duman, whom he met at Yale and married in 1988, and their two daughters, Katie Duman and Carolyn Duman. He will be dearly missed by those in the Division of Molecular Psychiatry, Yale, and by the entire scientific community.

